# Comparison of Glycemic Excursion Using Flash Continuous Glucose Monitoring in Patients with Type 2 Diabetes Mellitus Before and After Treatment with Voglibose

**DOI:** 10.1089/dia.2019.0484

**Published:** 2021-02-25

**Authors:** Selvam Kasthuri, Subramani Poongothai, Ranjit Mohan Anjana, Jayvel Selvakumar, Subramaniam Muthukumar, Sengottuevel Kayalvizhi, Syed Tariq, Evangelin Honey, Prasanna Kumar Gupta, Ulagamathesan Venkatesan, Viswanathan Mohan

**Affiliations:** ^1^ Madras Diabetes Research Foundation, Gopalapuram, Chennai.; ^2^ Dr. Mohan's Diabetes Specialities Centre & Madras Diabetes Research Foundation, Gopalapuram, Chennai.; ^3^ Dr. Mohan's Diabetes Specialities Centre, Tambaram East, Chennai.; ^4^ Dr. Mohan's Diabetes Specialities Centre, Anna Nagar, Chennai.; ^5^ Dr. Mohan's Diabetes Specialties Centre, Avadi, Chennai.; ^6^ Dr. Mohan's Diabetes Specialties Centre, Porur, Chennai.

**Keywords:** α-Glucosidase inhibitor, Flash glucose monitoring, FreeStyle Libre ProFlash, Glycemic variability, Postprandial hyperglycemia, Type-2 diabetes mellitus, Voglibose

## Abstract

***Purpose:*** To determine the effect of Voglibose add-on therapy on daily glycemic excursions (using FreeStyle^®^ Libre Pro™, a Flash glucose monitoring system) in Indian patients with type 2 diabetes mellitus (T2DM) receiving a stable dose of metformin (Met) or metformin+sulfonylurea (Met+SU).

***Patients and Methods:*** T2DM patients with glycosylated hemoglobin (HbA1c) ≥7.0% and at least two postprandial excursions ≥140 mg/dL (within 2 h of meal) during the screening phase (visit 1/day −14 ± 2) were enrolled in this prospective, multicenter interventional study. The patients were randomized at visit 2 (day 0 ± 2) to receive Voglibose 0.2 or 0.3 mg tablets (BID/TID) as add-on therapy to Met and Met+SU. All the patients were followed at day 14 ± 2 (visit 3), month 3 ± 14 days (visit 4), 14 weeks (i.e., visit 4 + 14 days) ±2 days (visit 5), and month 6 ± 14 days (visit 6). Continuous glucose monitoring was performed to study glycemic excursions at visits 2, 3, and 5. The study outcomes were: change in average number of glycemic excursions per day, percent time spent in glucose fluctuations, mean Postprandial glucose (PPG), Fasting plasma glucose (FPG), day and night time mean glucose levels from baseline to day 14 and week 14; change in mean amplitude of glycemic excursion (MAGE) from baseline to 14 weeks; and mean HbA1c level at 3 and 6 months.

***Results:*** Out of 110 patients enrolled, 101 patients (91.8%) (Met+SU+Voglibose: 73 and Met+Voglibose: 28) completed the study. There was a significant decrease in average number of glycemic excursions per day from baseline to day 14 in the Met+Sul+Voglibose group and to week 14 in the Met+Voglibose group. There was also a significant reduction in percent time spent above target glucose range from baseline to day 14 in both treatment groups and to week 14 in the Met+SU+Voglibose group. A significant reduction in mean PPG area under the curve, day and night time mean glucose levels, and mean FPG levels from baseline to day 14 was reported in both treatment groups. A significant reduction in night time glucose, and average MAGE and HbA1c levels was reported from baseline to week 14 in the Met+Voglibose group and the Met+SU+Voglibose group, respectively. At 6 months, body weight, glucose levels, cholesterol, low-density lipoprotein-cholesterol, and HbA1c were significantly lower, especially in the Met+SU+Voglibose arm.

***Conclusion:*** Voglibose was useful in reducing glycemic variability and improving glycemic control in Asian Indian adults with T2DM. (CTRI/2018/04/013074)

## Introduction

Diabetes mellitus (DM), especially type 2 DM (T2DM), is a growing global health concern and is a leading cause of morbidity and mortality worldwide.^1^ The significance of maintaining the glycemic control within target levels has been shown by various prospective trials on T2DM.^2,3^ The importance of lowering the postprandial glucose (PPG), fasting plasma glucose (FPG), and glycemic variability (GV) to achieve optimal glycemic control has also been well established.^4–6^

GV (i.e., intraday or day-to-day short-term fluctuations in blood glucose levels) imposes harmful effects on the arterial wall and is considered an important determinant of cardiovascular damage.^5,7–9^ The episodes of GV are largely contributed by the events of postprandial hyperglycemic excursions. It is believed that frequent and large glucose fluctuations and postprandial spikes in blood glucose lead to cardiovascular damage due to endothelial dysfunction, oxidative stress, and activation of inflammation and coagulation mechanisms, which results in penetration of lipoproteins into the arterial wall in patients with DM.^10,11^ Hence, lowering of GV and PPG levels in these patients can possibly prevent future cardiovascular events.

The mean amplitude of glucose excursion (MAGE) is considered the gold standard for estimating mealtime-related GV but its calculation requires glucose measurements in a continuous fashion. Continuous glucose monitoring (CGM) and flash glucose monitoring (FGM) provide continuous information on standard deviation (SD) and coefficient of variation, which helps in assessing the changes in GV over time.^11^ However, there are some challenges to use these devices in routine clinical management of diabetes. These include high cost of devices, need for recalibration, periodic replacement of sensors, day-to-day variability in glycemic patterns, lack of health care professionals trained in the use of CGM devices, and lack of standardization of software methods for analysis of CGM data.^12^

Alpha-glucosidase inhibitors (AGIs) are considered effective medications in reducing postprandial hyperglycemia.^13,14^ Indeed, they play a significant role in the Indian setting due to high carbohydrate consumption in India.^15^ The AGIs are used in various situations: as the first-line therapy in newly diagnosed T2DM patients insufficiently treated with diet and exercise alone and also in combination with oral anti-diabetic agents and insulin, if these drugs fail to achieve the target for glycosylated hemoglobin (HbA1c) and postprandial glucose.^14^ The AGIs are also used as a part of dual (along with metformin [Met] or other first-line agents) or triple therapy (along with Met+Sulphonylureas [SUs] or other second-line agents), if target glycemic goals are not achieved within the given timeframe.^4^ Studies have shown that AGIs significantly improve MAGE and minimize glycemic fluctuations by inhibiting carbohydrate digestion in the small intestine and delaying its absorption in T2DM patients.^16–20^

Voglibose, a potent competitive inhibitor of intestinal α-glucosidase, delays digestion and absorption of dietary polysaccharides, resulting in effective control of PPG excursions, HbA1c levels, and weight gain.^21–25^ Usually, Voglibose is administered as 0.2 mg thrice daily before each meal, but the dosage can be increased to 0.3 mg in cases of aggravated PPG response.^22,26^ Voglibose has been considered safe (due to poor absorption after oral use) and effective as a monotherapy or a combination therapy.^23,27–33^ In one study, Voglibose was found to be effective in lowering daily glycemic excursions, HbA1c, and plasma glucose levels and also in improving insulin sensitivity in T2DM patients.^34^

In clinical management, physicians usually prefer to prescribe combination therapies instead of a monotherapy, particularly if the target glycemic goal is not achieved with monotherapy. Met and Sulfonylureas (SU) are the most common oral antidiabetic therapies. In combination, metformin suppresses excess glucose production, and SU increases insulin secretion from the pancreas. Both agents effectively lower FPG and PPG. Since there is no literature available regarding the impact of a combination of Voglibose with Met or Met+SU on FPG, PPG, and HbA1c levels and intra-day glycemic excursions in T2DM patients, this study was taken up to assess the effects of voglibose as an add-on therapy, on the daily glycemic excursions (using FreeStyle^®^ Libre Pro™ Flash [a FGM device]; Abbott, Alameda, CA), in T2DM patients receiving a stable dose of Met or Met+SU.

## Materials and Methods

### Study participants

This was an investigator-initiated, multicenter, open-label, randomized, single-arm, prospective, interventional study conducted in T2DM patients aged 18–70 years (both inclusive), with body mass index (BMI) ≥23 kg/m^2^ and HbA1c >7.0% (for ≥3 months before screening). Patients who were on a stable dose of Met/Met+SU, had at least two postprandial excursions above 140 mg/dL (2 h after a meal) and compliance with using the CGM device during the 14 days before enrollment were included in this study. This study was conducted between April 2016 and September 2017 (CTRI/2018/04/013074). Patients were recruited from four centers in Chennai, India.

Patients with known hypersensitivity to α-glucosidase inhibitors, received voglibose, acarbose, or any other oral hypoglycemic agents (except Met and SU) for more than 14 days during the past 3 months; had renal dysfunction, congestive heart failure, and hepatic insufficiency; or a history of diabetic ketoacidosis, inflammatory bowel disease, colonic ulceration, partial intestinal obstruction, or alcohol/substance abuse were excluded from the study. Pregnant, lactating women and patients on insulin or glucagon-like peptide-1 were also not included in the study.

The study protocol was approved by Institutional Ethics Committee of the Madras Diabetes Research Foundation. The study was conducted in accordance with the principles of the Declaration of Helsinki, International Conference on Harmonization Good Clinical Practice (ICH GCP) guidelines, and Indian regulatory guidelines (Indian Council of Medical Research and Indian GCP guidelines). Written informed consent was obtained from all the participants.

### Study visits

The study consisted of 5 visits: visit 1 (screening phase; day −14 ± 2), visit 2 (randomization phase; day 0 ± 2), visit 3 (transition phase; day 14 ± 2), visit 4 (maintenance phase; month 3 ± 14 days), visit 5 (visit 4 + 14 days ±2; week 14), and visit 6 (follow-up phase; month 6 ± 14 days).

### Study intervention

After evaluating patient eligibility criteria, a FreeStyle Libre Pro Sensor (FGM system) was applied to the upper arm of all patients. HbA1c, lipid profile (total cholesterol, triglycerides, high-density lipoprotein [HDL]-cholesterol, low-density lipoprotein [LDL]-cholesterol, and non-HDL-cholesterol), FPG, and PPG levels were assessed at visit 1. During visit 2, 14-day CGM data were collected to analyze the baseline readings. Patients with at least two PPG excursions above 140 mg/dL (within 2 h of meal) were enrolled and randomized to receive voglibose (Abbott Healthcare Private Limited, India) 0.2 or 0.3 mg tablets, two or three times daily as an add-on therapy to Met/Met+SU. The FGM device was removed and the data were collected at visit 3, along with an assessment of HbA1c (through FGM), FPG, and PPG (laboratory method) levels. At visit 4, the FGM device was inserted, and the data were collected post-14 days at visit 5 along with measurement of HbA1c, FPG, and PPG levels. At visit 6, BMI, systolic and diastolic blood pressure, HbA1c, FPG, PPG, and lipid profile were assessed. A patient diary was dispensed to each patient at visit 1 to record their time of meal intake and any adverse events (AEs) during the conduct of the study.

### Outcome assessments

The primary outcome was the change in average number of glycemic excursions per day and percent time spent in glucose fluctuations from baseline to day 14 and week 14. The secondary outcomes were the changes in mean PPG area under the curve (within 3 h after each meal; breakfast, lunch, and dinner), day- and night-time mean glucose levels, and mean FPG (measured by CGM device and laboratory methods) from baseline to day 14 and week 14; change in MAGE from baseline to week 14; and change in HbA1c, FPG, and PPG level, lipid profile, BMI, and body weight from baseline to 3 and 6 months; and frequency of AE.

### Measurements and definitions

The FreeStyle Libre Pro sensor, an FGM device (inserted subcutaneously), consists of a reader and a sensor (inserted in the back of the upper arm), which measures glucose levels in interstitial fluid through a small (5 mm long, 0.4 mm wide) filament. The glucose readings are recorded every 15 min over 24 h, capturing up to 1344 glucose results for 14 consecutive days. The CGM allows to record daily glucose profile and glycemic excursions. On ambulatory glucose profile, glycemic excursions are represented as a variation in glucose reading from the mean or median glucose, the degree of up and down fluctuation (amplitude), and the frequency of variations.^35,36^ The interquartile range (25th and 75th percentile curves) on the glucose profile is a good indicator of the degree of glycemic excursions. The daily glucose summary report shows daily glucose, time in target, time below target (≤70 mg/dL), and time above target (≥140 mg/dL) within a selected time frame. Glycemic excursions/fluctuations are defined through FGM as hypoglycemia and hyperglycemia when the glucose readings are ≤70 and ≥140 mg/dL, respectively.

Day time and night time: Time between 6:00 AM and 10:00 PM is defined as day time, and 10:00 PM to 6:00 AM is defined as night time.

MAGE was calculated from day 0 to day 14, followed by month 3, to further 14 days by taking the arithmetic mean of blood glucose increases or decreases (from blood glucose nadirs to peaks or vice versa) when both ascending and descending segments exceeded the value of one SD of the blood glucose for the same 24-h period.^37^

### Statistical analysis

Considering a 10% dropout rate, it was planned to enroll 55 patients in the Met+SU+Voglibose group and 55 in the Met ± SU group. Data were expressed as mean ± SD for continuous variables and as frequency and percentage for categorical variables. The data between two different visits were compared by using paired *t*-test, and the data between the treatment groups were compared by using independent *t*-test. *P* < 0.05 was considered statistically significant. All analyses were performed by using SAS version 9.4.

## Results

A total of 110 patients (Met+Voglibose group: 31 patients; Met+SU+Voglibose group: 79 patients) were recruited in the study in each arm. Although we had originally planned to include 55 patients, in clinical practice, the group with Met, SU, and voglibose was more common and hence was not able to get enough patients in Met+Voglibose arm. However, this unequal distribution of numbers would not affect the results on glycemic parameters because the aim was to investigate the addition of voglibose and this is addressed separately in the two arms of the study.

Of the 110 patients enrolled, 101 (91.8%) completed the trial, including 28 (25.4%) patients in the Met+Voglibose group and 73 (72.3%) patients in the Met+SU+Voglibose group. Overall, 108 out of 110 patients (98.2%) were compliant with the study medication (Met+Vogl group: 31 patients; Met+SU+Voglibose: 77 patients).

### Baseline demographics and clinical characteristics

The mean (SD) age of the patients in the Met+Voglibose group was 49.8 (8.6) years and 50.9 (9.4) years in the Met+SU+Voglibose group. A total of 59 (54.6%) patients were men. The mean (SD) duration of DM was 5.9 (5.6) years. Other baseline demographics and clinical characteristics are summarized in Table 1.

**Table 1.** Baseline Characteristics of the Study Population

Parameter	Met+Voglibose (*n* = 31)	Met+SU+Voglibose (*n* = 77)	Total (*n* = 108)
Age (years), mean (SD)	49.8 (8.6)	50.9 (9.4)	50.6 (9.1)
Men	21 (67.7)	38 (49.3)	59 (54.6)
Women	10 (32.3)	39 (50.7)	49 (45.4)
BMI (kg/m^2^)	28.2 (4.1)	27.8 (4.2)	27.9 (4.1)
Duration of diabetes mellitus (in years)	3.4 (4.6)	6.9 (5.7)	5.9 (5.6)
Family history of diabetes mellitus, *n* (%)	23 (74.2)	59 (76.6)	82 (75.9)
Dyslipidemia	19 (61.3)	44 (57.1)	63 (58.3)
Neuropathy	13 (41.9)	27 (35.1)	40 (37.0)
Hypertension	8 (25.8)	24 (31.2)	32 (29.6)
Arthritis	1 (3.2)	1 (1.3)	2 (1.8)
CHD/IHD	1 (3.2)	1 (1.3)	2 (1.8)

All values are mean ± SD.

BMI, body mass index; CHD, coronary heart disease; IHD, ischemic heart disease; Met, metformin, SU, sulfonylurea.

### Primary outcome

Table 2 describes the average number of glycemic excursions (defined through CGM device as hypoglycemia [≤70 mg/dL] and hyperglycemia [≥180 mg/dL]) observed per day at day 14 and week 14. There was a statistically significant reduction in average number of glycemic excursions per day from baseline to day 14 in the Met+SU+Voglibose group (*P* = 0.01) and to week 14 in the Met+Voglibose group (*P* = 0.01).

**Table 2.** Average Number of Glycemic Excursions Observed Per Day at Baseline, Day 14, and Week 14 and Changes in Average Number of Glycemic Excursions from Baseline to Day 14 and Week 14

Statistics	Baseline visit		Day 14/visit 3		Week 14/visit 5	
	Met+Voglibose arm	Met+Sul+Voglibose arm	Met+Voglibose arm	Met+Sul+Voglibose arm	Met+Voglibose arm	Met+Sul+Voglibose arm
Mean (SD)	26.64 (14.32)	34.34 (22.47)	27.08 (21.61)	25.62 (14.76)	17.89 (11.03)	23.41 (18.87)
Range (min, max)	5.31, 49.53	6.54, 85.93	5.33, 61.29	7.42, 63.87	3.20, 37.40	5.10, 78.73
Mean Change(n)	—	—	−3.26	−8.02	−7.79	−10.40
*P*-value^*^	—	—	0.69	0.01	0.01	0.08

^*^ *P*-values were calculated by using paired *t* test at a 5% level of significance. *P*-value was a comparison between baseline visit and post-baseline visit.

Supplementary Table S1 shows that there was a significant increase in percent time spent within target glucose range in patients receiving Met+SU+Voglibose from baseline to day 14 (*P* < 0.0001) and week 14 (*P* = 0.0003), with corresponding reductions in percent time spent above target glucose range at day 14 (*P* = 0.0002) and week 14 (*P* = 0.0007). In the Met+Voglibose arm also, there was a significant increase in percent time spent within target glucose range from baseline to day 14 (*P* = 0.0143) and a significant decrease in percent time spent above target glucose range from baseline to day 14 (*P* = 0.001).

### Secondary outcome

Supplementary Table S2 shows that there was a statistically significant reduction in mean PPG during 3 h after each meal (breakfast, lunch, and dinner) from baseline to day 14 in both Met+SU+Voglibose and Met+Voglibose groups; however, no such significant decrease was noted in either of the treatment groups at week 14.

There was a significant reduction in day and night time mean glucose levels from baseline to day 14 in Met+SU+Voglibose and Met+Voglibose groups and a significant reduction in night time glucose levels from baseline to week 14 in the Met+Voglibose group (*P* = 0.03). There was a significant reduction in mean FPG (at least 10-h overnight fasting) from baseline to day 14 in Met+Voglibose (*P* = 0.02) and Met+SU+Voglibose (*P* = 0.02) groups (Supplementary Table S3).

**FIG. 1.**
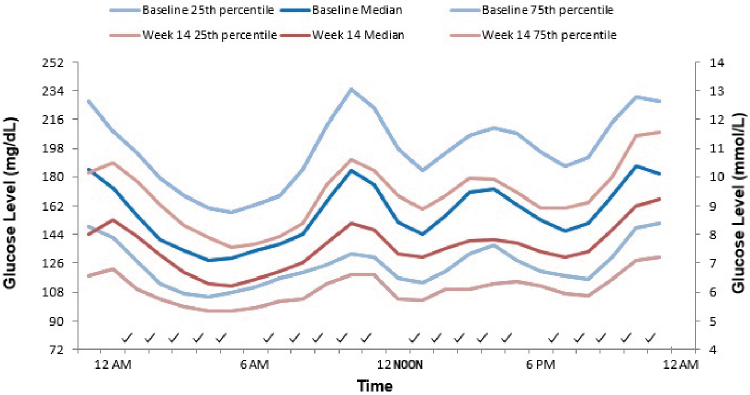
25th Percentile, median, and 75th percentile at baseline and after 14 weeks of treatment with voglibose.

**FIG. 2.**
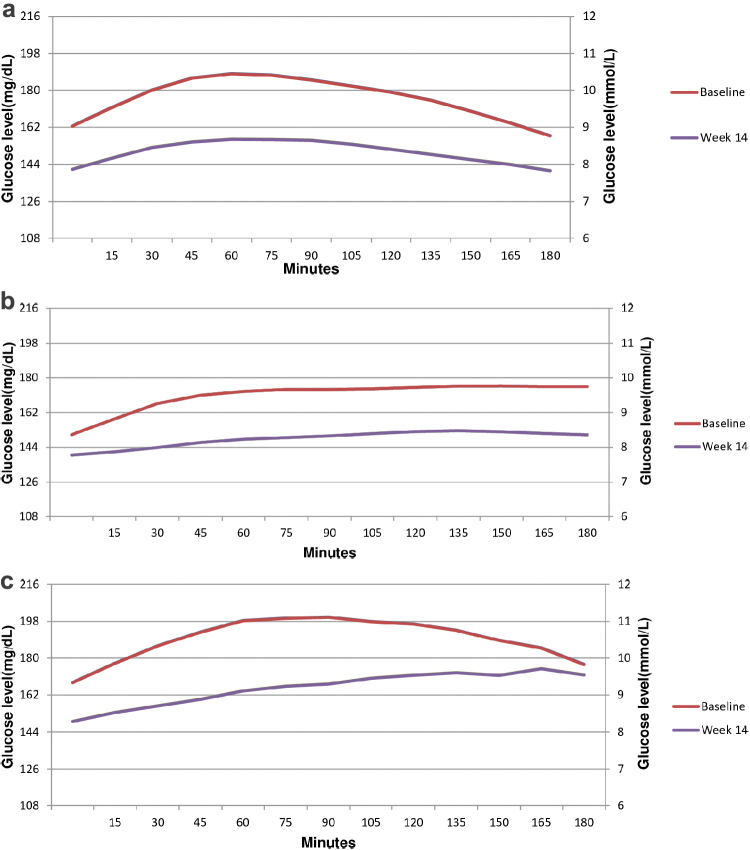
**(a)** Changes in mean postprandial glucose level during 3 h after breakfast from baseline to week 14. **(b)** Changes in mean postprandial glucose level during 3 h after lunch from baseline to week 14. **(c)** Changes in mean postprandial glucose level during 3 h after dinner from baseline to week 14.

Figure 1 depicts the average, along with the 25th and 75th percentile of 24-h glucose levels at baseline and at week 14 after administration of voglibose. Voglibose administration revealed a distinct effect as the average glucose levels were lower at week 14 as compared with baseline over the period of 24 h. Similar results were noted when 3-h postmeal (breakfast, lunch, and dinner) glucose patterns were evaluated at baseline and week 14 (Fig. 2a–c).

The changes in GV calculated as MAGE by using CGM and HbA1c are provided in Supplementary Table S4. There was a significant reduction in the average MAGE (*P* = 0.0128) and HbA1c (*P* = 0.0013) from baseline to week 14 in the Met+SU+Voglibose group only.

A significant reduction was also observed in FPG, PPG, HbA1c, BMI, body weight, systolic blood pressure, total cholesterol, triglycerides, LDL-cholesterol, and non-HDL cholesterol (except HDL-cholesterol) in the overall population from screening visit to month 6 (*P* ≤ 0.0026) (Supplementary Table S5).

Biochemical and clinical parameters such as weight, BMI, PPG, and triglycerides were significantly reduced at month 6 in both treatment groups.

Supplementary Table S6 shows that the levels of FPG, total cholesterol, LDL-cholesterol, and non-HDL-cholesterol were significantly reduced, especially in the Met+SU+Voglibose arm.

### Safety outcome

A total of 13 (12.0%) patients (Met+Vogl group: 4 [12.9%]; Met+SU+Voglibose group: 9 [11.7%]) reported at least one AE. In the Met+Voglibose group, one patient each had diarrhea, injury, upper respiratory tract infection, and skin infection. In the Met+SU+Voglibose group, two patients had worsening of hypertension, and one patient had gastritis, diarrhea, dyspepsia, hyperlipidemia, dermatitis, diabetic foot ulcer, and discoloration of nails. None of these AEs except the gastrointestinal one was considered to be related to the use of voglibose. No serious AEs were reported in the study.

## Discussion

Short-term glycemic oscillations and postprandial hyperglycemia are important in the pathogenesis of vascular complications and both should be considered as targets of glucose-lowering therapies, when managing T2DM.^8,38^ Unlike other glycemic target such as HbA1c, which reflect a long-term average plasma glucose, real-time glucose fluctuations through CGM can be used to investigate daily glycemic control, which is impractical to check through fingersticks.^39^ α-Glucosidase inhibitors have been considered as effective agents in reducing elevated levels of PPG.^14^ AGIs are also used as an add-on therapy to Met when the target glycemic levels are not achieved within 3 months.^40^

Voglibose, a competitive α-glucosidase inhibitor, delays the absorption of glucose, resulting in attenuation of PPG and glycemic excursions.^20,34,41–43^ Strict control of PPG glycemic excursions is clinically important, as it could reduce the risk of chronic diabetes complications. However, there is paucity of clinical data related to improvement in daily glycemic excursion with the use of voglibose in patients with T2DM.

In this study, the administration of voglibose showed a reduction in the average number of daily glycemic excursions from baseline to day 14 and week 14 in patients who were on a stable dose of Met+SU and Met only. The 24-h average glucose levels (in both treatment groups) and the average MAGE (in the Met+SU+Voglibose group) significantly improved at week 14. Our results are in concordance with the previous literature.^17,34^ Voglibose also significantly reduced percent time spent above the glucose target range at day 14 (in both treatment groups) and week 14 (in the Met+SU+voglibose group). Similar findings were reported in another study.^44^

Various studies have reported the beneficial role of voglibose on PPG level both as a monotherapy and in combination with other oral antidiabetic drugs.^45–47^ Our study also presents a similar finding. In a multicenter, randomized, double-blind trial, acarbose significantly lowered HbA1c, FPG, PPG, and insulin levels and was found to be safe and well tolerated in T2DM patients who were inadequately controlled with diet and Met.^48^ These studies support the use of α-glucosidase inhibitors as a potential therapeutic option in patients with high PPG level or those who are poorly controlled with other hypoglycemic agents.

Further, we found that over the 6-month treatment with voglibose, there was a significant reduction in the levels of several lipid parameters. Insulin sensitivity is reported to be an essential and independent determinant of serum triglycerides and free fatty acid concentrations, suggesting an association between the decline in triglyceride levels and improvement in insulin sensitivity in voglibose-treated patients.^49^ The significant reduction in body weight, BMI, and SBP at 6 months further demonstrates the beneficial role of voglibose. In a previous study, voglibose treatment prevented the weight-increasing effect of pioglitazone in T2DM patients.^50^ Further, the combination therapy of acarbose and SU significantly lowered body weight in comparison to SU treatment alone (*P* < 0.001).^51^ These studies suggest that α-glucosidase inhibitors may serve as potentially useful drugs in terms of reducing body weight. However, this needs to be proven in future well-designed studies. The reduction in SBP may enhance the cardio-protective effects of voglibose but this is not fully established in clinical studies.

Though voglibose is poorly absorbed after oral administration, gastrointestinal (GI) tract-related side effects are the most commonly reported side-effects.^26,48^ In our study also, GI related side-effects were the most common. Previous studies suggest that voglibose treatment had significantly less adverse drug reactions compared with acarbose (56.7% vs. 90%, *P* < 0.05), a lower frequency of flatulence (56.7% vs. 90%), and abdominal distension (10% vs. 16.7%). This may be due to a lower dosage of voglibose used in comparison to acarbose dosage (0.2 mg vs. 100 mg thrice daily with meals for 8 weeks) or less digestive enzyme inhibition by voglibose.^21^ In our study, no serious AEs were reported, showing that the combination of voglibose with Met/Met+SU is a safe and well-tolerated regimen in patients with T2DM.

Our study has multiple strengths. First, it is one of the first to prospectively evaluate the add-on effect of voglibose on multiple glycemic markers. Second, we have also followed patients over a period of 6 months. Third, we have assessed multiple parameters, including average number of variations, percent time spent in glucose fluctuations, and change in MAGE to capture GV over the period of 14 weeks. Fourth, the impact of voglibose was not only studied on glycemic control but also studied on lipid parameters, body weight, and BMI. However, the study has a few limitations as well. First, there was no control group. Second, we had unequal numbers of participants in the two arms. Third, we could not assess other metabolic indices that may affect the glycemic profile, such as the C peptide, Glucagon-Like peptide-1 (GLP-1), glucose-dependent insulinotropic peptide, and glucagon levels. Finally, we did not check the insulin secretion or its sensitivity to understand the possible mechanistic effect of adding voglibose to existing antidiabetic drugs such as Met and SU.

## Conclusion

Voglibose therapy in T2DM patients previously receiving MET alone or in combination with an SU for 14 weeks was effective in lowering FPG and HbA1c, in reducing blood glucose levels throughout the entire 24-h period, and in reducing GV (MAGE and postprandial excursions). In addition, voglibose therapy was associated with an increase in time spent within the target glucose range (70–180 mg/dL) (3.9–10.0 mmol/L), with no increase in hypoglycemia and no weight gain. Finally, voglibose use up to 6 months was associated with lower weight, BMI, glucose levels, and HbA1c, especially in the Met+SU+Voglibose arm. Thus, voglibose can be considered as a useful, safe, and effective add-on therapy in T2DM management. However, large controlled prospective clinical trials are warranted to study the long-term effects of voglibose along with other antidiabetic therapies in T2DM management.

## Supplementary Material

Supplemental data

Supplemental data

Supplemental data

Supplemental data

Supplemental data

Supplemental data
