# Supplementary material for: Comparison of Glycemic Excursion Using Flash Continuous Glucose Monitoring in Patients with Type 2 Diabetes Mellitus Before and After Treatment with Voglibose
Source: Diabetes Technol Ther. 2021 Feb 25;23(3):213–20. doi: 10.1089/dia.2019.0484 (PMC7906864; doi:10.1089/dia.2019.0484)
Supplement: Supplemental data [file Supp_TableS1.docx]

**Supplementary Table 1: Mean change in time spent within target glucose range, time below target glucose range, and time above target glucose range from baseline to day 14 and week 14**

|  | **Baseline Visit** | | | **Day 14/Visit 3** | | | **Week 14/Visit 5** | | |
| --- | --- | --- | --- | --- | --- | --- | --- | --- | --- |
| **Statistics** | **Overall** | **Met+Voglibose Arm** | **Met+SU+Voglibose Arm** | **Overall** | **Met+Voglibose Arm** | **Met+SU+Voglibose Arm** | **Overall** | **Met+Voglibose Arm** | **Met+SU+Voglibose Arm** |
| **Time Spent Within Target Glucose Range (%)** | | | | | | | | | |
| Mean Change | - | - | - | 8.57 | 10.11 | 8.08 | 8.99 | 7.12 | 9.70 |
| P-value* | - | - | - | <0.0001 | 0.01 | <0.0001 | < 0.0001 | 0.14 | 0.0003 |
| **Time Spent Below Target Glucose Range(%)** | | | | | | | | | |
| Mean Change | - | - | - | 1.5 | 3.11 | 1.01 | 1.52 | 0.44 | 1.92 |
| P-value* | - | - | - | 0.2021 | 0.39 | 0.25 | 0.2694 | 0.89 | 0.19 |
| **Time Spent Above Target Glucose Range(%)** | | | | | | | | | |
| Mean Change | - | - | - | -10.07 | -13.21 | -9.10 | -10.51 | -7.56 | -11.62 |
| P-value* | - | - | - | <.0001 | 0.001 | 0.0002 | 0.0006 | 0.26 | 0.0007 |

Met, Metformin; SU, Sulfonylurea

Note: *P-values were calculated using paired t test at 5% level of significance. P-value was a comparison between Baseline visit and Post baseline visit.
