# Supplementary material for: Comparison of Glycemic Excursion Using Flash Continuous Glucose Monitoring in Patients with Type 2 Diabetes Mellitus Before and After Treatment with Voglibose
Source: Diabetes Technol Ther. 2021 Feb 25;23(3):213–20. doi: 10.1089/dia.2019.0484 (PMC7906864; doi:10.1089/dia.2019.0484)
Supplement: Supplemental data [file Supp_TableS2.docx]

**Supplementary Table 2: Changes in mean postprandial glucose level during 3 hours following breakfast, lunch, and dinner from baseline to day 14 and week 14**

|  | **Baseline Visit** | | | **Day 14/Visit 3** | | | **Week 14/Visit 5** | | |
| --- | --- | --- | --- | --- | --- | --- | --- | --- | --- |
| **Statistics** | **Overall** | **Met+Voglibose Arm** | **Met+SU+Voglibose Arm** | **Overall** | **Met+Voglibose Arm** | **Met+SU+Voglibose Arm** | **Overall** | **Met+Voglibose Arm** | **Met+SU+Voglibose Arm** |
| **Mean postprandial glucose level during 3 hours following breakfast** | | | | | | | | | |
| Mean Change | - | - | - | -20.90 | -34.52 | -17.98 | -29.12 | -28.87 | -29.27 |
| P-value* | - | - | - | 0.0008 | 0.006 | 0.01 | 0.0072 | 0.06 | 0.06 |
| **Mean postprandial glucose level during 3 hours following lunch** | | | | | | | | | |
| Mean Change | - | - | - | -19.20 | -20.56 | -20.01 | -22.62 | -14.45 | -27.64 |
| P-value* | - | - | - | 0.0010 | 0.101 | 0.004 | 0.0306 | 0.21 | 0.08 |
| **Mean postprandial glucose level during 3 hours following dinner** | | | | | | | | | |
| Mean Change | - | - | - | -27.56 | -42.39 | -23.15 | -27.07 | -25.92 | -27.77 |
| P-value* | - | - | - | 0.0002 | 0.008 | 0.006 | 0.0128 | 0.11 | 0.06 |

Met, Metformin; SU, Sulfonylurea

Note: *P-values were calculated using paired t test at 5% level of significance. P-value was a comparison between baseline visit and post baseline visit.
