# Supplementary material for: Comparison of Glycemic Excursion Using Flash Continuous Glucose Monitoring in Patients with Type 2 Diabetes Mellitus Before and After Treatment with Voglibose
Source: Diabetes Technol Ther. 2021 Feb 25;23(3):213–20. doi: 10.1089/dia.2019.0484 (PMC7906864; doi:10.1089/dia.2019.0484)
Supplement: Supplemental data [file Supp_TableS3.docx]

**Supplementary Table 3: Changes in day and night time mean glucose level and mean FPG level from baseline to day 14 and week 14**

|  | **Baseline Visit** | | | **Day 14/Visit 3** | | | **Week 14/Visit 5** | | |
| --- | --- | --- | --- | --- | --- | --- | --- | --- | --- |
| **Statistics** | **Overall** | **Met+Voglibose Arm** | **Met+SU+Voglibose Arm** | **Overall** | **Met+Voglibose Arm** | **Met+SU+Voglibose Arm** | **Overall** | **Met+Voglibose Arm** | **Met+SU+Voglibose Arm** |
| **Day time mean glucose level** | | | | | | | | | |
| Mean change | - | - | - | -18.06 | -26.16 | -16.42 | -22.36 | -17.76 | -24.24 |
| P-value* | - | - | - | 0.001 | 0.02 | 0.01 | 0.01 | 0.10 | 0.05 |
| **Night time mean glucose level** | | | | | | | | | |
| Mean change | - | - | - | -18.9 | -26.50 | -17.84 | -18.19 | -19.42 | -17.69 |
| P-value* | - | - | - | 0.001 | 0.02 | 0.01 | 0.03 | 0.03 | 0.12 |
| **Mean FPG (as Measured by continuous glucose monitoring device and laboratory method)** | | | | | | | | | |
| Mean change | - | - | - | -16.60 | -25.85 | -14.22 | -17.69 | -17.75 | -17.65 |
| P-value* | - | - | - | 0.002 | 0.02 | 0.02 | 0.04 | 0.12 | 0.16 |

FPG, Fasting plasma glucose; Met, Metformin; SU, Sulfonylurea

Note: *P-values were calculated using paired t test at 5% level of significance. P-value was a comparison between baseline visit and post baseline visit.
