# Supplementary material for: Comparison of Glycemic Excursion Using Flash Continuous Glucose Monitoring in Patients with Type 2 Diabetes Mellitus Before and After Treatment with Voglibose
Source: Diabetes Technol Ther. 2021 Feb 25;23(3):213–20. doi: 10.1089/dia.2019.0484 (PMC7906864; doi:10.1089/dia.2019.0484)
Supplement: Supplemental data [file Supp_TableS4.docx]

**Supplementary Table 4: Mean changes in MAGE and HbA1c from baseline to week 14**

| **Statistics** | **Baseline Visit** | | **Week 14/Visit 5** | |
| --- | --- | --- | --- | --- |
|  | **Met+Voglibose (n=28)** | **Met+SU+Voglibose (n=73)** | **Met+Voglibose (n=25)** | **Met+SU+Voglibose (n=66)** |
| **MAGE** | | | | |
| Mean (SD)  SE  Range (min, max) | 54.71 (28.06)  5.30  20.00, 134.00 | 81.18 (46.47)  5.44  24.00, 280.00 | 62.44 (48.50)  9.70  20.00, 266.00 | 63.27 (33.59)  4.13  10.00, 172.00 |
| Mean Change (n) |  |  | 4.88 (25) | -14.76 (66) |
| Percent Change |  |  | 14.12 | -22.06 |
| 95% CI of Mean Change |  |  | -17.45, 27.21 | -26.27, -3.24 |
| *P-value |  |  | 0.65 | 0.01 |
| #P-value |  |  |  | 0.92 |
| **HbA1c** | | | | |
| Mean (SD)  Range (min, max) | 6.96 (1.21)  4.20, 9.60 | 7.46 (1.42)  5.00, 12.00 | 6.49 (0.96)  4.90, 8.60 | 6.88 (1.34)  4.10, 11.80 |
| Mean Change |  |  | -0.45 | -0.59 |
| Percent Change |  |  | -6.74 | -7.74 |
| 95% CI of Mean Change |  |  | -1.05:0.16 | -0.95:-0.24 |
| *P-value |  |  | 0.13 | 0.001 |
| #P-value |  |  |  | 0.18 |

CI, Confidence interval; HbA1c, glycosylated hemoglobin; MAGE, Mean amplitude of glycemic excursion; Met, Metformin; SD, Standard deviation; SE, Standard error; SU, Sulfonylurea

Note: *P-Values were calculated using paired t test at 5% level of significance. P-value is comparison between Baseline visit and Post baseline visit.

#P-Values were calculated using Independent T test at 5% level of significance. P-value is comparison between two treatment groups.
