# Supplementary material for: Comparison of Glycemic Excursion Using Flash Continuous Glucose Monitoring in Patients with Type 2 Diabetes Mellitus Before and After Treatment with Voglibose
Source: Diabetes Technol Ther. 2021 Feb 25;23(3):213–20. doi: 10.1089/dia.2019.0484 (PMC7906864; doi:10.1089/dia.2019.0484)
Supplement: Supplemental data [file Supp_TableS5.docx]

**Supplementary Table 5: Biochemical parameters at screening visit and month 6**

| **Variables** | **ScreeningVisit**  **n=100** | **Month 6/Visit 6**  **n=100** | **P-value** |
| --- | --- | --- | --- |
| Age (years) | 50.4±9.3 | - | - |
| Male n (%) | 54 (54) | - | - |
| Weight (kg) | 71.2±12.8 | 70.2±12.9 | 0.001 |
| BMI(kg/m^2^) | 28.0±4.1 | 27.6±4.3 | <0.001 |
| Systolic blood pressure (mmHg) | 125±13 | 121±10 | 0.002 |
| Diastolic blood pressure (mmHg) | 78±7 | 77±6 | 0.16 |
| HbA1c (%) | 8.9±1.4 | 7.9±1.1 | <0.001 |
| Fasting plasma glucose (mg/dL) | 171±52 | 156±41 | 0.001 |
| Post-prandial plasma glucose(mg/dL) | 279±71 | 241±59 | <0.001 |
| Total cholesterol (mg/dL) | 180±37 | 166±37 | 0.0002 |
| Triglycerides (mg/dL) | 154±66 | 134±56 | 0.0003 |
| HDL-cholesterol (mg/dL) | 39±9 | 39±9 | 0.96 |
| LDL-cholesterol (mg/dL) | 110±33 | 100±32 | 0.001 |
| Non-HDL-cholesterol (mg/dL) | 139±37 | 126±34 | 0.002 |

BMI, body mass index; HbA1c, glycosylated hemoglobin; HDL, high density lipoprotein; LDL, low density lipoprotein; Met, Metformin; SU, Sulfonylurea

Note:P-values were calculated using paired t test between the visits at 5% level of significance.
