# Supplementary material for: Comparison of Glycemic Excursion Using Flash Continuous Glucose Monitoring in Patients with Type 2 Diabetes Mellitus Before and After Treatment with Voglibose
Source: Diabetes Technol Ther. 2021 Feb 25;23(3):213–20. doi: 10.1089/dia.2019.0484 (PMC7906864; doi:10.1089/dia.2019.0484)
Supplement: Supplemental data [file Supp_TableS6.docx]

**Supplementary Table 6: Biochemical parameters at screening visit and 6 months after voglibose add-on therapy in subgroups**

| **Variables** | **ScreeningVisit** | | **Month 6/Visit 6** | | **P-value** | |
| --- | --- | --- | --- | --- | --- | --- |
|  | **Met+ Voglibose (n=28)** | **Met+SU+ Voglibose (n=73)** | **Met+ Voglibose (n=28)** | **Met+SU+ Voglibose (n=73)** | **Met+ Voglibose** | **Met+SU+ Voglibose** |
| Weight (kg) | 73.01 (13.95) | 70.27 (12.32) | 71.84 (14.74) | 69.36 (12.16) | 0.03 | 0.001 |
| BMI(kg/m^2^) | 28.03 (4.12) | 27.92  (4.23) | 27.55  (4.34) | 27.56  (4.24) | 0.02 | 0.001 |
| Fasting plasma glucose (mg/dL) | 146.68 (34.51) | 182.42 (53.41) | 136.39 (26.99) | 164.00 (42.81) | 0.10 | 0.003 |
| Post-prandial plasma glucose(mg/dL) | 259.54 (72.06) | 289.26 (69.05) | 221.86 (61.66) | 247.34 (57.05) | 0.01 | <.0001 |
| Total Cholesterol (mg/dL) | 169.57 (36.43) | 185.03 (36.76) | 161.14 (34.24) | 168.05 (37.72) | 0.26 | 0.0001 |
| Triglycerides (mg/dL) | 161.86 (69.67) | 153.83 (64.49) | 134.93 (65.19) | 135.08 (51.57) | 0.01 | 0.007 |
| HDL-Cholesterol (mg/dL) | 37.14 (9.13) | 40.07 (8.66) | 37.93 (8.86) | 39.77 (8.28) | 0.58 | 0.81 |
| LDL-Cholesterol (mg/dL) | 100.36 (34.83) | 114.10 (31.35) | 96.21 (27.05) | 101.34 (33.23) | 0.54 | 0.0005 |
| Non-HDL-Cholesterol (mg/dL) | 130.57 (36.68) | 142.31 (38.06) | 123.21 (32.19) | 128.29 (34.94) | 0.34 | 0.003 |

BMI, body mass index; HDL, high density lipoprotein; LDL, low density lipoprotein; Met, Metformin; SU, Sulfonylurea

Note:P-values were calculated using paired t test between the visits at 5% level of significance.
